# Association between epicardial adipose tissue and transfusion-dependent β-thalassemia

**DOI:** 10.1007/s10554-025-03535-2

**Published:** 2025-10-09

**Authors:** Michele Malagù, Federico Marchini, Filomena Longo, Marco Zuin, Giovanni Orazio, Andrea Capanni, Maria Letizia Berloni, Federica Frascaro, Elisabetta Tonet, Alberto Cossu, Martina Culcasi, Beatrice Bonsi, Cristina Balla, Francesco Vitali, Matteo Bertini

**Affiliations:** 1 Cardiology Unit, Azienda Ospedaliero-Universitaria di Ferrara, Via Aldo Moro 8, Ferrara, 44124 Italy; 2 Day Hospital Thalassemia and Hemoglobinopathies, Azienda Ospedaliero- Universitaria di Ferrara, Ferrara, Italy; 3 Radiology Unit, Azienda Ospedaliero-Universitaria di Ferrara, Ferrara, Italy

**Keywords:** Hemoglobinopathy, Epicardial fat, Atrial fibrillation, Arrhythmia, Cardiac, Magnetic resonance

## Abstract

**Background:**

Epicardial adipose tissue (EAT) is a fat depot with secretive and neuromodulative properties, involved in the pathogenesis of cardiovascular diseases. Transfusion-dependent β-thalassemia (TDβT) is an inherited disorder characterized by chronic anemia and at risk for the development of several comorbidities. Patients with TDβT have a higher incidence of cardiovascular diseases compared to the general population.

**Methods:**

The aim of this study was to evaluate EAT thickness in a cohort of patients with TDβT (study group), compared to age- and sex-balanced patients with no TDβT (control group). Secondary endpoint included evaluation of differences in the regional distribution of EAT between patients with or without cardiovascular disease (NCT05508932).

**Results:**

Overall, 272 patients were enrolled, of whom 136 in study group and 136 in control group. EAT thickness was significantly higher in the study group at left atrium, left atrioventricular groove and right atrioventricular groove compared to control group (4.2 vs. 3.0, 12.2 vs. 10.0 and 13.0 vs. 11.0 mm, respectively, *p* < 0.05 for all). Multivariable analysis confirmed an independent association between EAT and TDβT. The regional distribution of EAT showed significant differences between patients with or without atrial fibrillation, diabetes mellitus, stroke or heart failure.

**Conclusions:**

Patients with TDβT have higher values of EAT compared to controls with no TDβT.

**Graphical abstract:**

Central illustration

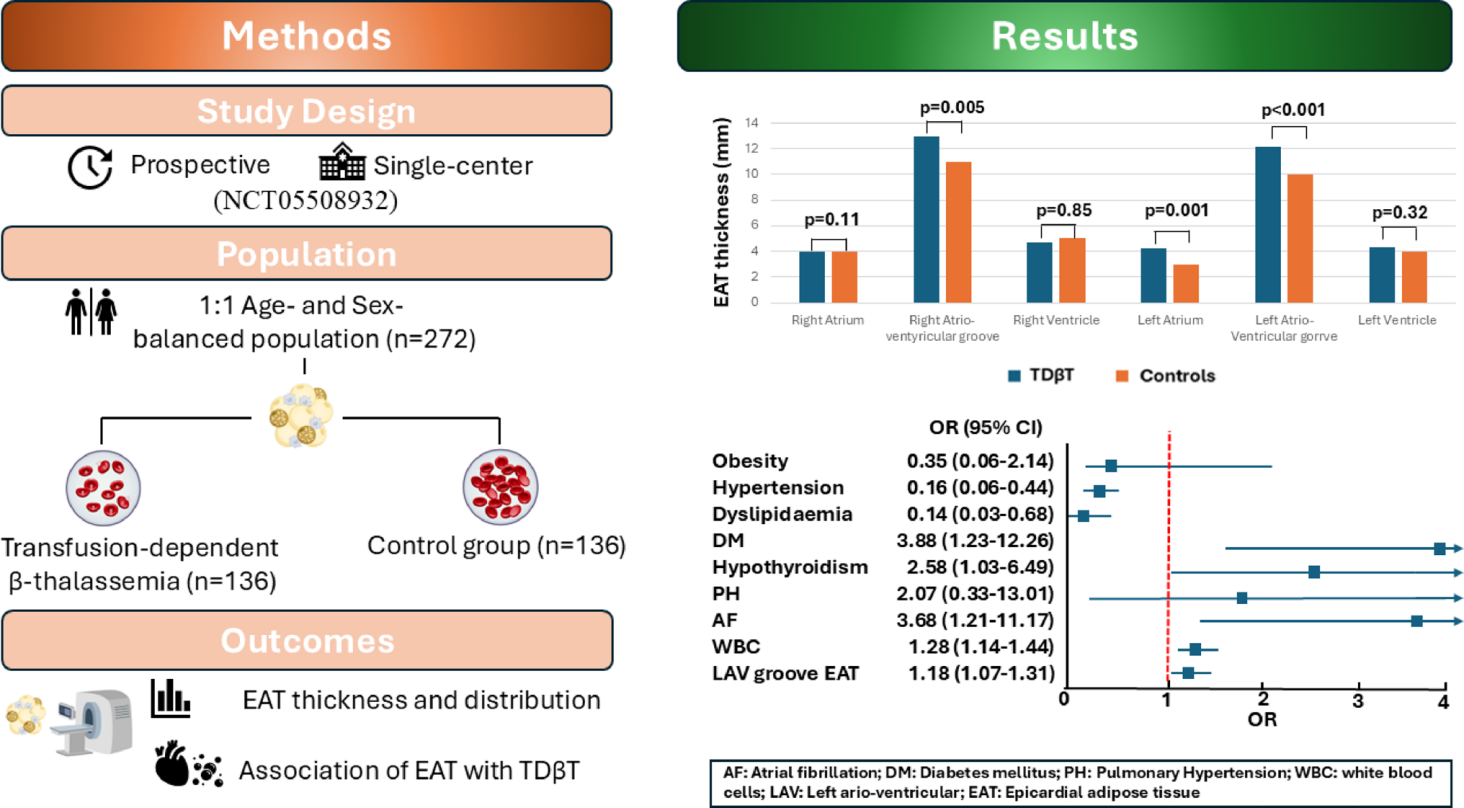

## Introduction

β-thalassemia is a chronic disease characterized by a genetic defect in hemoglobin chain synthesis, resulting in ineffective erythropoiesis and anemia [1]. While some patients may be asymptomatic, subjects with severe forms of β-thalassemia require lifelong red blood cells transfusions for survival. Patients affected by transfusion dependent β-thalassemia (TDβT) typically experience growth and developmental delay, compensatory extramedullary marrow expansions, peripheral hemolysis, coagulation anomalies, iron overload and multiple organ failure [2].The more frequent comorbidities that may develop include heart failure, atrial fibrillation (AF), diabetes mellitus, stroke, pulmonary hypertension, liver cirrhosis, hypothyroidism, osteoporosis and cancer [3–5]. Heart disease currently remains the leading cause of death in these patients [4].

Epicardial adipose tissue (EAT) is a fat depot located between the myocardium and the visceral pericardium [6]. Recent studies have highlighted the role of EAT in the pathophysiology of different chronic diseases, identifying EAT as potential modifiable risk factor and a biomarker involved in the pathogenesis of heart failure, AF, coronary artery disease, diabetes mellitus and stroke [7–10]. The harmful effects of EAT are primarily mediated by the secretion of proinflammatory and profibrotic cytokines, as well as by neuromodulation, which directly affect the heart due to the absence of an anatomical barrier between EAT and the adjacent myocardium [8]. Differences in the regional distribution of EAT may have an impact over the risk of developing different cardiovascular chronic conditions [8]. The growing interest in EAT is also due to the fact that a reliable measurement is now possible with noninvasive imaging techniques [11].

Interestingly, several diseases which are frequently observed in patients with TDβT, such as heart failure, AF, diabetes mellitus and stroke, are also conditions in which EAT has a recognized pathogenetic role [4, 5, 8]. This observation led to the question whether EAT in thalassemia may be different than in the general population and which role it may play in the pathophysiology of those diseases. Currently, no studies have explored the potential link between EAT and TDβT. The aim of this study was to explore whether EAT in thalassemia may be different than in the general population.

## Methods

The main purpose of the present study was to assess EAT in a cohort of patients affected by TDβT, compared to controls with no history of thalassemia. Secondary objectives were to explore differences in the regional distribution of EAT between subgroups of patients with and without cardiovascular diseases.

β-THAL (“*Atrial fibrillation in β-thalassemia*”) is a prospective, single-center, observational study conducted at the *Azienda Ospedaliero-Universitaria di Ferrara*, Italy. The study was registered on *ClinicalTrials.gov* (NCT05508932). Study protocol was approved by the local Ethics Committee (*Comitato Etico Indipendente di Area Vasta Emilia Centro*) and all patients signed informed consent. Inclusion criteria were: (i) confirmed TDβT, and (ii) age ≥ 18 years. Exclusion criteria were: (i) state of pregnancy, and (ii) inability to give informed consent. All patients underwent routine cardiological examination and cardiac magnetic resonance imaging (MRI) as the standard of care on an annual basis [12]. The first MRI examinations after enrollment were considered as the index MRI for the present analysis.

As control group, patients with no history of β-thalassemia, who underwent cardiac MRI scan over study period, were considered. We created a balanced selection of patients in control group based on sex and age using a stratified random sampling method. Patients were grouped into strata based on sex and age categories (10-year intervals). Within each stratum, we assigned random values and selected an equal number of patients to ensure that the distribution of sex and age in control group matched study group population.

All MRI imaging procedures were performed with a 1.5 T scanner (Siemens, Magnetom Aera) using a standard protocol. All images were obtained during breath holding at expiration. The cine images included the acquisition of long axis slice (two- and four-chamber) and a stack of short-axis slices, covering the whole left ventricle using a balanced steady-state free precession sequence. For myocardial iron overload assessment, three slices (basal, medium and apical) were acquired using T2* gradient multiecho sequences. For hepatic iron overload evaluation, a mid-hepatic slice was obtained. Post-processing analysis was performed using a commercially available software (IntelliSpace Portal, Philips Healthcare). EAT measurements were performed in steady state free precession sequences, assessing thickness at six distinct locations: right ventricular (RV) free wall, right atrioventricular groove, right atrial (RA) free wall, left ventricular (LV) free wall, left atrioventricular groove, left atrial (LA) free wall. Measurements were performed in standardized transversal four chamber and short axis views, when appropriate. EAT assessment at MRI was performed by two investigators blinded to patient clinical history. In case of disagreement, a third investigator was consulted, and the final decision was made by consensus.

Data regarding medical history, laboratory tests, drug therapy and imaging examinations were collected. AF was defined in accordance to the European Society of Cardiology guidelines [13]. Heart failure was defined as left ventricular ejection fraction (EF) ≤ 40% or previous hospitalization with clinical diagnosis of heart failure [14]. Chronic kidney failure (CKD) was defined as glomerular filtration rate (GFR) < 60 ml/min/1.73 m^2^ for 3 months or more, irrespective of cause. Cardiac iron overload was defined as T2* <20 ms at MRI scan [15].

### Statistical analysis

Categorical variables were presented as frequencies and percentages. Continuous variables were expressed as mean ± standard deviation if normally distributed, or as median and interquartile range (IQR), as determined by the Shapiro-Wilk test. Differences between groups were analysed using Pearson *χ*^2^, Spearman, Student *t* or Mann-Whitney *U* test, where appropriate. A multivariable logistic regression model was performed to investigate the association between TDβT and other variables. Specifically, variables with a p value < 0.10 in the univariate analysis were further evaluated in a stepwise logistic regression model. The results of the multivariate analysis are reported as odds ratio (OR) with 95% confidence interval (CI). A two-tailed p value of < 0.05 was considered statistically significant. Statistical analyses were conducted using SPSS software, version 25 (IBM Corp, Armonk, NY, USA).

## Results

Between September 2022 and September 2024, 136 patients with TDβT were enrolled (study group). One-hundred and eighty-four patients with no history of TDβT were screened as controls, of whom 136, balanced by age and sex to patients in study group, were included in the final analysis (control group).

Patient characteristics are shown in Table 1. The prevalence of arterial hypertension and dyslipidaemia was significantly lower in the study group, compared to the control group. Conversely, patients in study group had a higher prevalence of AF, diabetes mellitus, hypothyroidism, pulmonary hypertension and splenectomy. The prevalence of heart failure and stroke was relatively low, although slightly higher in the study group, and the difference between groups did not reach statistical significance. White blood cells (WBC) were higher in study group. A total of 46 patients had a history of AF, of whom 38 out of 136 in the study group and 8 out of 136 in the control group (27.9% vs. 5.9%, *p* < 0.001). The characteristics of AF and the differences between groups are presented in Table 2. No significant differences were found between groups regarding the characteristics of AF.

**Table 1:** Clinical characteristics of the population

Variable	Overall (*n* = 272)	Study group (*n* = 136)	Control group (*n* = 136)	*p* value
Age (years)	51 [43–57]	50 [45–55]	51 [43–57]	0.36
Male sex	135 (49.6%)	66 (48.5%)	69 (50.7%)	0.80
Obesity	17 (6.3%)	5 (3.7%)	12 (8.8%)	0.08
Arterial hypertension	59 (21.7%)	14 (10.3%)	45 (33.1%)	**< 0.001**
Dyslipidaemia	31 (11.4%)	2 (1.5%)	29 (21.3%)	**< 0.001**
Smoking habit				0.39
- Active	33 (12.1%)	19 (14.0%)	14 (10.3%)	
- Previous	39 (14.3%)	22 (16.2%)	17 (12.5%)	
Diabetes mellitus	37 (13.6%)	27 (19.8%)	10 (7.4%)	**0.004**
Hypothyroidism	58 (21.3%)	40 (29.4%)	18 (13.2%)	**0.002**
Previous stroke	4 (1.5%)	3 (2.2%)	1 (0.7%)	0.62
Pulmonary hypertension	12 (4.4%)	10 (7.3%)	2 (1.5%)	**0.034**
Splenectomy	93 (34.2%)	93 (68.4%)	0 (0%)	**< 0.001**
COPD	5 (1.8%)	3 (2.2%)	2 (1.5%)	1
Renal failure	5 (1.8%)	2 (1.5%)	3 (2.2%)	1
Heart failure	7 (2.6%)	5 (3.7%)	2 (1.5%)	0.45
Atrial fibrillation	46 (16.9%)	38 (27.9%)	8 (5.9%)	**< 0.001**
White blood cells (U x 10^3^/µl)	7.9 [6.0–10.0]	9.4 [7.1–12.6]	6.9 [5.2–8.9]	**< 0.001**
LV diastolic volume index (ml/m^2^)	80 [69–94]	80 [72–95]	80 [68–92]	0.29
LV ejection fraction %	63 [60–67]	64 [61–68]	62 [60–65]	0.75
RV diastolic volume index (ml/m^2^)	76 [63–89]	76 [65–93]	76 [62–88]	0.18
RV ejection fraction (%)	63 [59–67]	63 [58–68]	64 [60–67]	0.80

BMI: body mass index. COPD: chronic obstructive pulmonary disease. LV: left ventricle. RV: right ventricle

**Table 2:** Characteristics of patients with atrial fibrillation

Variable	OveralL (*n* = 46)	Study group (*n* = 38)	Control group (*n* = 8)	*p* value
Time from diagnosis (years)	9 [3–16]	9 [3–17]	13 [4–18]	0.96
Type				0.90
- Paroxysmal	30 (65.2%)	25 (65.8%)	5 (62.5%)	
- Persistent	9 (19.6%)	7 (18.4%)	2 (25%)	
- Permanent	7 (15.2%)	6 (15.8%)	1 (12.5%)	
CHA_2_DS_2_VASc score				0.56
- 0	18 (39.1%)	16 (42.1%)	2 (25%)	
- 1	12 (26.1%)	9 (23.7%)	3 (37.5%)	
- 2	10 (21.7%)	9 (23.7%)	1 (12.5%)	
- 3	4 (8.7%)	3 (7.9%)	1 (12.5%)	
- ≥ 4	2 (4.3%)	1 (2.6%)	1 (12.5%)	
EHRA symptom scale				0.18
- 1	29 (63%)	24 (63.2%)	5 (62.5%)	
- 2a	10 (21.7%)	9 (23.7%)	1 (12.5%)	
- 2b	3 (6.5%)	2 (5.3%)	1 (12.5%)	
- 3	3 (6.5%)	3 (7.9%)	0 (0%)	
- 4	1 (2.2%)	0 (0%)	1 (12.5%)	
Cardioversion	29 (63%)	26 (68.4%)	3 (37.5%)	0.13
Transcatheter ablation	12 (26.1%)	10 (26.3%)	2 (25%)	1
Antiarrhythmic therapy	34 (73.9%)	30 (78.9%)	4 (50%)	0.18
Anticoagulant therapy	33 (71.7%)	28 (73.7%)	5 (62.5%)	0.67

EHRA: European Heart Rhythm Association

### Study group

All patients in study group were treated with periodic red blood cells transfusions, with a median inter-transfusion interval of 15 days (IQR 15–21). Additionally, all individuals underwent regular iron chelation therapy (deferoxamine 50/136, 36.8%, deferasirox 58/136, 42.6%, and deferiprone 32/136, 23.5%). At the index cardiac MRI, 5 patients had a septal T2* value of < 20 ms (3.7%), with four of these patients also having a global T2* value of < 20 ms. A median of seven previous cardiac MRI were available per patient over the preceding 12 years. At previous cardiac MRI scans, 25/136 patients (18.4%) had at least one finding of T2* <20 ms in any myocardial segment, with a median time of eight years between the most recent finding and enrolment. The liver iron concentration was 2.78 (IQR 1.44–5.16) mg Fe/g dry weight. None of the patients had a history of liver cirrhosis. The median ferritin level was 504 (IQR 313–773) ng/ml.

### Eat

**Fig. 1:**
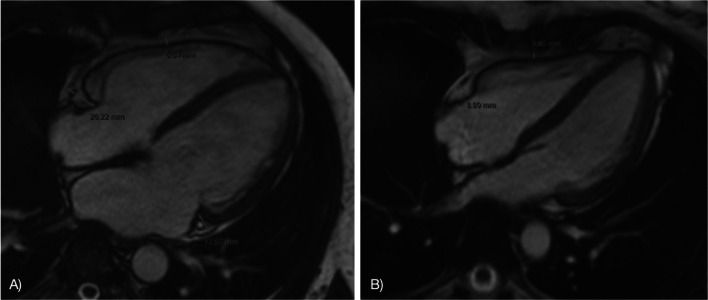
Epicardial adipose tissue at magnetic resonance imaging. (**A**) Patient with transfusion-dependent β-thalassemia. (**B**) Patient with no history of thalassemia

Patients in study group exhibited significantly higher EAT values, compared to control group, at three specific locations: the left atrium, the left atrioventricular groove and the right atrioventricular groove (Table 3; Fig. 1). No significant differences in EAT thickness were observed between the two groups at the right atrium, right ventricle and left ventricle.

**Table 3:** Thickness of epicardial adipose tissue at different locations

Location	Overall (*n* = 272)	Study group (*n* = 136)	Control group (*n* = 136)	*p* value
Right atrium	4.0 [3.0–4.81]	4.0 [3.3–4.7]	4.0 [3.0–5.0]	0.11
Right atrio-ventricular groove	12.3 [10.0–15.0]	13.0 [11.0–15.0]	11.0 [9.0–14.5]	**0.005**
Right ventricle	4.7 [3.5–6.0]	4.7 [3.6–5.9]	5.0 [3.0–7.0]	0.85
Left atrium	4.0 [3.0–5.0]	4.2 [3.4–5.4]	3.0 [3.0–4.0]	**0.001**
Left atrio-ventricular groove	11.0 [9.0–13.0]	12.2 [10.6–13.5]	10.0 [8.0–12.0]	**< 0.001**
Left ventricle	4.0 [3.0–5.0]	4.3 [3.2–5.2]	4.0 [3.0–5.0]	0.32

Values are expressed in mm

When comparing all subjects with a history of AF to subjects with no history of AF, EAT resulted significantly higher at right atrium and left atrium, but not at the other locations (Table 4). Analysis of regional distribution of EAT in patients with and without history of diabetes mellitus, stroke and heart failure, revealed additional differences. Patients with diabetes mellitus had significantly higher EAT values at the right atrium and at the left ventricle. In patients with a history of stroke, compared to patients with no history of stroke, EAT was lower at the left ventricle. In contrast, patients with heart failure had higher EAT at both the right atrium and the left atrium (Table 4).

**Table 4:** Differences in regional distribution of epicardial adipose tissue between patients with or without history of atrial fibrillation, diabetes mellitus, stroke and heart failure

Location	AF (*n* = 46)	No AF (*n* = 226)	*p* value	DM (*n* = 37)	No DM (*n* = 235)	*p* value	Stroke (*n* = 4)	No stroke (*n* = 268)	*p* values	HF (*n* = 7)	No HF (*n* = 265)	*p* value
Right atrium	4.4 [3.5–5.0]	3.9 [3.0–4.7]	**0.037**	4.2 [3.6–4.9]	3.9 [3.0–4.8]	**0.026**	3.4 [2.3–4.2]	4.0 [3.0–4.9]	0.42	5.4 [4.0–6.5]	4.0 [3.0–4.8]	**0.022**
Right atrio-ventricular groove	13.0 [10.2–16.1]	12.3 [10.0–14.7]	0.43	13.0 [11.0–15.9]	12.2 [10.0–14.8]	0.14	12.0 [6.9–16.2]	12.3 [10.0–15.0]	0.75	12.0 [11.0–16.6]	12.3 [10.0–15.0]	0.47
Right ventricle	5.2 [4.0–6.4]	4.5 [3.4–6.0]	0.09	4.7 [3.4–6.2]	4.7 [3.5–6.0]	0.83	3.7 [2.7–6.3]	4.8 [3.5–6.0]	0.36	4.6 [4.0–5.5]	4.7 [3.4–6.0]	0.89
Left atrium	5.0 [4.0–6.0]	3.9 [3.0–5.0]	**< 0.001**	4.0 [3.1–5.6]	4.0 [3.0–5.0]	0.32	3.0 [3.0 3.4]	4.0 [3.0–5.0]	0.29	5.3 [4.5–6.0]	4.0 [3.0–5.0]	**0.045**
Left atrio-ventricular groove	12.0 [9.5–13.8]	11.0 [9.0–13.0]	0.14	12.0 [10.7–13.1]	11.0 [9.0–13.0]	0.17	11.3 [6.8–11.6]	11.0 [9.0–13.0]	0.54	10.6 [9.3–13.0]	11.0 [9.0–13.0]	0.80
Left ventricle	4.5 [4.0–5.0]	4.0 [3.0–5.1]	0.30	4.5 [3.6–6.0]	4.0 [3.0–5.0]	**0.034**	2.9 [2.7–3.0]	4.0 [3.0–5.0]	**0.020**	5.0 [3.2–5.9]	4.0 [3.0–5.0]	0.36

Values are expressed in mm. AF: atrial fibrillation. DM: diabetes mellitus. HF: heart failure

The correlation between EAT and laboratory and imaging parameters is shown in Table 5.

**Table 5:** Correlation between EAT and laboratory/imaging parameters

EAT location	WBC		T2*		LV diastolic volume index		RV diastolic volume index	
	*r*	*p*	*r*	*p*	*r*	*p*	*r*	*p*
RA	0.08	0.23	0.01	0.90	−0.04	0.56	−0.05	0.49
RAV groove	0.15	0.02	−0.03	0.75	−0.11	0.09	−0.07	0.25
RV	0.12	0.07	0.14	0.13	−0.10	0.13	−0.08	0.21
LA	0.15	0.03	−0.01	0.89	−0.01	0.85	−0.02	0.80
LAV groove	0.14	0.03	0.1	0.26	−0.01	0.96	−0.09	0.17
LV	0.10	0.09	0.04	0.66	−0.10	0.13	−0.08	0.22

RA: right atrium. RAV: right atrioventricular. RV: right ventricle. LA: left atrium. LAV: left atrioventricular. LV: left ventricle. WBC: white blood cells

### Univariate and multivariate analysis

Univariate analysis revealed that obesity, diabetes mellitus, hypothyroidism, pulmonary hypertension, AF, WBC, and EAT at the left atrioventricular groove were associated with TDβT, whereas arterial hypertension and dyslipidaemia were inversely associated with TDβT (Table 6). The multivariate analysis confirmed the independent association of TDβT with diabetes mellitus, hypothyroidism, AF, WBC, and EAT at the left atrioventricular groove (Table 6). Additionally, arterial hypertension and dyslipidaemia were confirmed to be significantly less prevalent in TDβT patients.

**Table 6:** Univariate and multivariate analysis for association with transfusion-dependent β-thalassemia

Variable	Univariate			Multivariate		
	Odds ratio	95% CI	*p* value	Odds ratio	95% CI	*p* value
Obesity	0.39	0.14–1.15	0.089	0.35	0.06–2.14	0.25
Arterial hypertension	0.19	0.10–0.36	< 0.001	0.16	0.06–0.44	**< 0.001**
Dyslipidaemia	0.05	0.011–0.20	< 0.001	0.14	0.03–0.68	**0.015**
Diabetes mellitus	3.12	1.45–6.74	0.004	3.88	1.23–12.26	**0.021**
Hypothyroidism	2.73	1.47–5.07	0.001	2.58	1.03–6.49	**0.043**
Pulmonary hypertension	5.32	1.14–24.74	0.033	2.07	0.33–13.01	0.44
Atrial fibrillation	6.20	2.77–13.90	< 0.001	3.68	1.21–11.17	**0.021**
White blood cells	1.35	1.22–1.49	< 0.001	1.28	1.14–1.44	**< 0.001**
LAV groove EAT	1.17	1.07–1.27	< 0.001	1.16	1.04–1.29	**0.006**

CI: confidence interval. BMI: body mass index. LAV: left atrio-ventricular. EAT: epicardial adipose tissue

## Discussion

The main findings of our study are as follows:

- EAT was significantly higher in patients with TDβT than in controls.
- Differences were found in the regional distribution of EAT between patients with and without AF, diabetes mellitus, stroke and heart failure.

To the best of our knowledge, this is the first study comparing thalassemic patients and non-thalassemic controls focused on EAT. While the role of EAT in cardiovascular disease pathophysiology is well established, the association between EAT and TDβT is a novel finding that raises important new questions and hypotheses. Per se, patients with thalassemia are at increased risk of developing comorbidities such as AF, diabetes mellitus, stroke and heart failure, all of which share EAT as a common risk factor. Furthermore, patients with TDβT have a higher prevalence of other comorbidities, like hypothyroidism and pulmonary hypertension, for which the role of EAT has not been explored. The presence of multiple baseline differences between the study group and control group shouldn’t be surprising as this reflects the complex health conditions of patients with thalassemia [4]. In our results, the multivariate analysis confirmed the independent association between EAT and TDβT. However, understanding the pathogenetic role of EAT in this context exceeds the scope of our study, but our results represent important findings that will need further investigations.

Moreover, our results showed that AF, hypothyroidism and diabetes mellitus were independently associated with TDβT. The prevalence of AF in our study group was 27.9%. This finding aligns with recent studies among AF in thalassemia, which reported prevalence rates of 14–29% [16-19]. These rates of AF among patients with thalassemia are impressively higher than the 2–4% documented in the general population [13]. Historically, cardiac iron overload was a primary cause of arrhythmias in TDβT patients [20, 21]. However, with the advent of regular iron chelation therapy, the natural history of the disease has changed. Recent prospective studies conducted in contemporary cohorts, specifically investigating the relationship between arrhythmias and iron, found that AF was unrelated to iron overload [18, 22]. Confirming this, in our study patients with cardiac iron overload at the most recent MRI were only 3.7%. A combination of factors underlies the higher prevalence of AF in thalassemic patients, among which a role is played by anemia, inflammation, chronically elevated cardiac output, atrial fibrosis and remodeling [2]. In addition to those factors, EAT may play a role. The independent association between EAT and AF, which is well established in the general population, has been observed and confirmed in patients with TDβT in a previous observational study conducted by our research group [18]. However, a major limitation of that study was the absence of a control group to be compared with thalassemic patients. Our current work addresses that limitation and enhances our understanding of EAT role in this setting.

Hypothyroidism and diabetes mellitus are among the most common comorbidities in thalassemia, a finding also confirmed by our study population [4]. Conversely, we observed a significantly lower prevalence of arterial hypertension in patients with TDβT. In the general population, arterial hypertension is one of the most common risk factors associated with AF [23]. This observation is not unexpected, as the mechanisms underlying the development of AF in thalassemia are, at least in part, different from the general population [2, 4]. It can be hypothesized that, in the context of β-thalassemia, arterial hypertension may play a lesser role in the development of AF compared to other factors. However, further studies could provide additional understanding of those observations.

Our results showed differences in the regional distribution of EAT between patients with and without TDβT. Specifically, thalassemic patients showed higher EAT values at the left atrium and at both atrio-ventricular grooves. Previous studies have suggested that the regional distribution of EAT may influence the development of various diseases such as AF, heart failure or coronary artery disease. For example, only EAT at the left atrium was associated with AF, while pericoronary EAT was associated with coronary artery disease [8, 24]. EAT affects the adjacent myocardium through the secretion of cytokines that promote inflammation (e.g., IL-6, tumor necrosis factor) and fibrosis (e.g., activin A, tissue growth factor, matrix metalloproteinases), as well as through the infiltration of free fatty acids and autonomic disorders via ganglionated plexi. At the atria, one consequence of these effects is AF. Notably, our study found that EAT in TDβT patients was higher at both atrioventricular grooves and at the left atrium, but not at the right atrium. When considering AF, diabetes mellitus, stroke and heart failure, no differences were observed at the atrioventricular grooves. Moreover, the association between EAT at left atrioventricular groove and TDβT was independent from other variables. We could hypothesize that the higher distribution of EAT at the left and right atrioventricular grooves may be characteristic of thalassemia. Other parameters, such as the fat attenuation index assessed by cardiac CT could be useful in order to better understand two main points: the synergistic effect of EAT and inflammation on onset and chronicity of AF, the influence of thalassemia on inflammation of the pericardial fat. Further studies could provide additional insights into this finding.

## Limitations

Our research has several limitations. First, it is a single-center study, which may limit the applicability to other regional settings due to variations in treatment approaches worldwide. These variations include differences in access to regular blood transfusions and iron chelation therapy, particularly in developing countries. The control group consisted of patients who underwent cardiac MRI for various reasons and may not be fully representative of the general population. However, the availability of MRI data was a necessary condition for our analysis. Finally, since the primary aim of our study was to investigate the association between EAT and TDβT, we were not able to provide any insights into potential causative relationships between these factors.

## Conclusions

In this cohort study, EAT was higher in patients with TDβT compared to age- and sex-balanced controls with no history of TDβT. Additionally, among these patients, those with AF, diabetes mellitus, or heart failure had higher EAT values than those without these conditions. Finally, the differences in the regional distribution of EAT may highlight distinct phenotypic patterns of the disease.

## Data Availability

The data underlying this article will be shared on reasonable request to the corresponding author.
